# Making the Case for Research on Disease-Modifying Treatments to Tackle Post-lingual Progressive Sensorineural Hearing Loss

**DOI:** 10.3389/fneur.2020.00290

**Published:** 2020-04-21

**Authors:** Vincent Van Rompaey

**Affiliations:** ^1^Department of Otorhinolaryngology and Head & Neck Surgery, Antwerp University Hospital, Edegem, Belgium; ^2^Department of Translational Neurosciences, Faculty of Medicine and Health Sciences, University of Antwerp, Antwerp, Belgium

**Keywords:** DFNA9, sensorineural hearing loss, bilateral vestibulopathy, hypothetical scenario, gene therapy

## Abstract

Hearing loss not only has a significant impact on the quality of life of patients and society, but its correlation with cognitive decline in an aging population will also increase the risk of incident dementia. While current management of hearing loss is focused on hearing rehabilitation (and essentially symptomatic), patients are suffering from the burden of progressive hearing loss before hearing aids or cochlear implants are fitted. Although these devices have a significant effect on speech understanding, they do not always lead to normal speech understanding, especially in noisy environments. A significant number of patients suffer from autosomal dominantly inherited disorders that can produce progressive sensorineural hearing loss. This includes DFNA9, a disorder caused by pathologic variants in the *COCH* gene that leads to post-lingual profound sensorineural hearing loss and bilateral vestibulopathy. Carriers of a pathogenic variant leading to DFNA9 can be diagnosed at the pre-symptomatic or early symptomatic stage which creates a window of opportunity for treatment. Preventing hearing loss from occurring or stabilizing progression would provide the opportunity to avoid hearing aids or cochlear implants and would be able to reduce the increased incidence of dementia. While innovative therapies for restoration of hearing have been studied for restoration of hearing in case of severe-to-profound sensorineural hearing loss and congenital hearing loss, further research is needed to study how we can modify disease progression in late-onset autosomal dominant hereditary sensorineural hearing loss. Recently, gene editing strategies have been explored in autosomal dominant disorders to disrupt dominant mutations selectively without affecting wild-type alleles.

“*Why the model of autosomal dominant hereditary hearing loss can be of interest.”*

## Introduction

Hearing loss has a significant impact on quality of life and society in general. Hearing impairment is the most frequent sensory deficit, affecting 360 million people worldwide and therefore it has been listed by the World Health Organization (WHO) as one of the priority diseases for research into therapeutic interventions to address public health needs (1). The global annual cost of unaddressed hearing loss has been estimated by the WHO to be in the range of $750–790 billion (1). Currently, no disease-modifying therapies are available to slow down or prevent progressive sensorineural hearing loss from happening. Instead, treatment is currently focused on hearing rehabilitation, which means fitting hearing aids that amplify sounds in case of moderate-to-severe sensorineural hearing loss (2, 3). In case of severe-to-profound sensorineural hearing loss, when amplification no longer leads to adequate speech perception, cochlear implantation may provide a solution (4–6). During cochlear implantation a multi-channel electrode is surgically introduced in the cochlea to electrically stimulate the spiral ganglion neurons, replacing mechano-transduction in the hair cells (7).

## Rehabilitation of Hearing Loss

Hearing aids and cochlear implants should be regarded as symptomatic treatments that are able to restore functionality (i.e., communication) and quality of life to a certain level. Bilateral cochlear implantation has been reported to restore binaural hearing to a certain level and improve speech comprehension, while reducing tinnitus burden and psychological comorbidities (8–10). However, in most health services, adult cochlear implantation is only reimbursed in a single ear because of its significant cost (10, 11). Despite the availability of these treatments, hearing-impaired patients are often reluctant to adopt any (12, 13). Although many studies have confirmed significant improvement in speech understanding after cochlear implantation, even in aging patients, many potential adult CI candidates are unaware of this treatment option or opt out, with <10% of those with severe-to-profound bilateral sensorineural hearing loss receiving a cochlear implant (14). This low rate is widespread, regardless of geographical location, and is independent of how health services are organized and country-specific economic output (15–18). In contrast, congenital severe-to-profound sensorineural hearing loss is identified at a very young age using universal neonatal hearing screening and the cochlear implantation rate in children is high (19). The obvious disadvantage of hearing rehabilitation is that we are waiting for loss of functionality to occur and act whenever certain hearing thresholds are reached to reimburse hearing aids or cochlear implants (6, 20).

## Hearing Loss and Cognitive Decline are Linked

Over the past decade the correlation between hearing loss and cognitive decline in the older population has gained more research interest. Large prospective studies have found an independent relationship between hearing loss on the one hand and age-related cognitive decline and incident dementia on the other hand (21–24). Worldwide, around 50 million people have dementia, with 10 million new cases added every year, and no cure available yet. Therefore, the WHO has also recognized dementia as a public health priority to identify any disease-modifying treatments (25, 26). Because of its close relationship to the cochlea, the vestibular system has also been implicated in cognitive decline (27). However, because earlier studies did not take a potentially concomitant sensorineural hearing loss in to account or were performed in a static condition, further research will be necessary to identify its role (28–32).

## The Pathophysiology Behind the Link Between Hearing Loss and Cognitive Decline

The underlying cause of the correlation between hearing and cognition remains unclear, but several hypotheses have been suggested. The *common cause hypothesis* suggests that hearing impairment and cognitive decline may result from one common mechanism. The *cognitive load on perception hypothesis* implies that a reduction in cognitive functioning may result in a heavier load on sensory processing. Both hypotheses suggest a top-down correlation. Another hypothesis, the *sensory-deprivation hypothesis*, implicates peripheral hearing loss as the direct cause of permanent cognitive decline, while the *information-degradation hypothesis* will point at depression and social isolation resulting from sensorineural hearing loss as the indirect cause. The latter hypotheses rather suggest a bottom-up causality from the periphery. At a pathophysiological level, evidence from rodent studies suggests that sensorineural hearing loss may result in a decreased adult hippocampal neurogenesis, which subsequently leads to impairments of learning and memory (33–35). At a clinical level, brain atrophy has been observed in longitudinal MRI studies in patients with hearing loss when compared with their normally hearing peers (36, 37). This brain atrophy may be a result of ongoing decreased adult hippocampal neurogenesis and would argue in favor of a bottom-up causality, i.e., peripheral sensorineural hearing loss induces changes in the central nervous system. Cognitive decline may lead to mild cognitive impairment, which may subsequently progress to incident dementia. Hearing loss has been identified as the most important modifiable risk factor to dementia onset in middle life (45–65 years). Livingston et al. raised the importance of prevention in dementia and focuses on potentially modifiable risk factors, which totals 35% of known risk factors (38). When specifically looking at these population attributable fractions, hearing loss accounts for 9.1% of these potentially modifiable risk factors, in contrast to hypertension (2%) and obesity (0.8%) (38).

## CAN Hearing Rehabilitation Slow Down Cognitive Decline?

The increased risk of cognitive decline in hearing-impaired patients brings the impact of progressive sensorineural hearing loss to another level. In case of mild hearing loss, the risk of incident dementia will increase by x1.89, it will increase by x3 where the patient progresses to moderate hearing loss and will increase by 4.94 if the patient reaches the level of severe-to-profound hearing loss (21). As hearing aids can improve hearing and contribute to reestablishing the individual's participation in society, they could have a positive effect on the expected trajectory of cognition (39). However, the results of studies investigating the effect of hearing aids on cognitive function in older adults are inconclusive (40–47). Recent studies have also studied the impact of unilateral cochlear implantation on the cognitive capabilities of older adults with bilateral severe-to-profound sensorineural hearing loss (28, 48–61). While cochlear implantation leads to a significant improvement of speech understanding in older adults and some improvement in cognition overall when compared to the preoperative performance, even experienced cochlear implant users still underperform when compared with their peers when matched for age, sex and education. It is unclear what the exact reason for some of the improvement that has been observed may be: are hearing aids and cochlear implants improving cognitive abilities or are cognitively healthy individuals more educated about hearing rehabilitation and more prone to seek help (62)?

## Challenges and Opportunities for Disease-Modifying Therapies In Progressive Post-Lingual Sensorineural Hearing Loss

**Figure 1:**
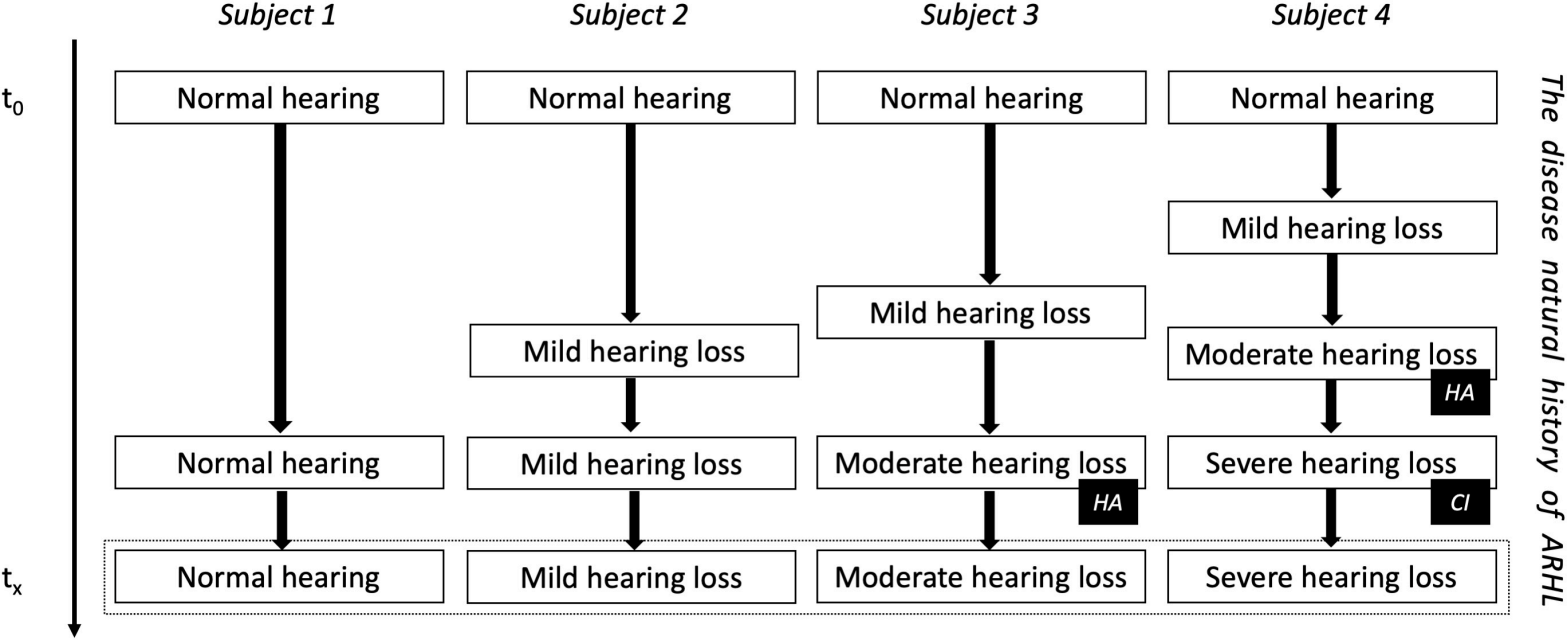
The natural evolution of age-related hearing loss (ARHL). Subject four is the example of DFNA9 in carriers of the P51S pathogenic variant in the *COCH* gene. HA, indication for a hearing aid; CI, indication for a cochlear implant.

Instead of waiting for hearing loss to occur and rehabilitate hearing function to a subpar level, we could aim to develop disease-modifying treatments that are able to slow down or prevent hearing loss progression. Current actions that try to prevent hearing loss are protection from noise exposure and adequate treatment of (or immunization to prevent) upper airway and central nervous infections, such as acute and chronic otitis media, or meningitis (1). A significant challenge in case of age-related hearing loss is the prediction of hearing loss progression. Although the annual deterioration rate of hearing is ~1 dB hearing level (dBHL) per year and patients with mild-to-moderate sensorineural hearing loss may progress to severe-to-profound sensorineural hearing loss, this progression may stabilize spontaneously for an indefinite period at any level (Figure 1) (63–65). For this reason, longitudinal studies evaluating hearing loss over time are essential to enable multi-state modeling and predictions of hearing progression on a population and individual level (66). Several potential therapeutic targets related to age-related hearing loss have been identified, including oxidative phosphorylation dysfunction-related apoptosis and mutations in mitochrondrial DNA. Strategies that have been suggested to influence hearing loss progression are stem cell-based therapy, gene therapy, aspirin, antioxidant defense, antioxidant enhancement, aldosterone modulation, and operant training (67–69). To date, no treatment has emerged to act as a disease-modifier to slow down or prevent sensorineural hearing loss.

There is one important cause of postlingual (adult-onset) sensorineural hearing loss where we do know what is happening in the cochlea and what will happen to hearing loss progression in a single adult patient: non-syndromic autosomal dominant hereditary hearing loss. Non-syndromic hearing impairment is a partial or total impairment of hearing not associated with other signs and symptoms. Between 75 and 80 percent of these cases are inherited through an autosomal recessive pattern, while another 20–25 percent of non-syndromic hearing impairment have an autosomal dominant pattern of inheritance (70, 71). The latter means that one copy of the altered gene in each cell is sufficient to cause the phenotype. Consequently, the inheritance rate is 50%. Most reported disorders with postlingual non-syndromic sensorineural demonstrate an autosomal dominant inheritance pattern. One of the first autosomal dominant disorders to be reported is called *DFNA9*. The DFN is an acronym for DeaFNess, while the A stands for autosomal dominant. DFNA9 is caused by heterozygous gain-of-function mutations or pathogenic variants in the *COCH* gene. To date, over 25 different variants have been identified worldwide (28). The *P51S* variant (DFNA9^P51S^) is the most frequently reported pathogenic variant in Belgium and the Netherlands. The phenotype is characterized by a progressive sensorineural hearing loss, starting from the 3rd−4th decade, followed by a rapid decline to severe-to-profound sensorineural hearing loss by the 6th−7th decade (72–74). Progressive vestibular dysfunction starts at a similar age and evolves toward bilateral vestibular function loss (bilateral vestibulopathy, BVP). BVP causes oscillopsia and imbalance while walking (especially in the dark) (75–83). Currently, no treatment is available to prevent or slow down sensorineural hearing loss or BVP in DFNA9 patients.

## Innovative Therapies to Treat Sensorineural Hearing Loss are Emerging

Over 100 clinical trials that evaluate novel inner ear therapies are ongoing worldwide, while only one of these trials involves gene therapy. The latter is the first-in-human phase 1/2 clinical trial (supported by the FDA) to upregulate the atonal gene (ATOH1/MATH1) in supporting cells of the inner ear and to trigger trans-differentiation into functional hair cells (84). For a review on the current state-of-art on gene therapy for human sensorineural hearing loss, please refer to the following papers (85, 86). Recently reported rodent studies on gene editing have generally been aiming to restore hearing in case of congenital sensorineural hearing loss by recovery of gene and protein expression, and subsequent restoration of sensory cell function, e.g., in Usher type 1c (87) or Usher type 1g (88). Clinically, this strategy would imply early treatment (i.e., the intrauterine or neonatal period in humans) in a population with prelingual sensorineural hearing loss, in contrast to adult-onset progressive sensorineural hearing loss. Recently, gene editing strategies have been explored in autosomal dominant disorders (which mainly involve single nucleotide substitutions) to disrupt dominant mutations selectively without affecting wild-type alleles (89). Specifically in DFNA36, *Tmc1* point mutations were targeted using an adeno-associated virus-mediated delivery to prevent deafness up to 1 year post-injection in mice (90). A similar strategy may work for other forms of autosomal dominant disorders (91). RNA inhibition using antisense nucleotides is another strategy that has been studied to prevent sensorineural hearing loss in Usher syndrome (92, 93).

## Why DFNA9 Can Serve as a Model for Age-Related Hearing Loss

The nature of DFNA9 may present some opportunities for developing a disease-modifying treatment for progressive sensorineural hearing loss and testing this potential treatment in a future clinical trial. Not only do we know the exact cause at a protein level (i.e., a mutated isoform of cochlin), we also know in what area in the inner ear the protein is expressed most abundantly (i.e., fibrocytes of the spiral ligament and spiral limbus) and what its pathophysiology is (primarily fibrocyte degeneration and subsequent spiral ganglion degeneration) (94). Earlier studies have shed light on the annual deterioration rate in DFNA9^P51S^, i.e., 3 dBHL per year -in contrast to 1 dBHL per year in age-related hearing loss- and has demonstrated high penetrance, i.e., the occurrence of a phenotype in pathogenic variant carriers (74). It is important to notice that each pathogenic variant in DFNA9 may have another phenotype.

In potential pathogenic variant carriers with a known family history, we can establish the genetic diagnosis in pre-symptomatic patients by taking a routine blood sample. If tested at the age of 18 years, early diagnosis in normal hearing carriers will lead to a significant therapeutic interval of up to 25 years to administer any disease-modifying therapy. It also provides opportunity to study if any such therapy will be able to reduce progression speed or (in an optimal scenario) prevent hearing loss from occurring at all. Although restoring hearing up to a certain level has become mainstream because of cochlear implantation, little is known on patient attitudes toward preventing, stabilizing or slowing down progression of sensorineural hearing loss by means of such future potentially disease-modifying treatments. In a survey performed in 53 carriers of pathogenic variants in the *COCH* gene, various hypothetical scenarios were presented while using a Likert scale to study willingness to participate in clinical trials studying potential treatment strategies (95). Overall, most symptomatic patients would likely consider participation in future innovative inner ear therapy trials, even if it would only slow down the decline of hearing and vestibular function. However, they were more equivocal on high-risk treatments or a placebo-controlled study design. Next to DFNA9, currently over 40 genes are known to be responsible for autosomal dominant non-syndromic hearing loss and may present similar opportunities for innovative treatment. The former data can be used to inform the recruitment and consent process into future innovative treatments to treat these other autosomal dominant disorders. However, the phenotype of different autosomal dominant disorders can vary quite significantly, and therefore may have a significant impact on patient anticipation.

## Data Availability Statement

All datasets generated for this study are included in the article/supplementary material.

## Author Contributions

VV designed the work, drafted the manuscript, and approves for publication of the content and agrees to be accountable for all aspects of the work in ensuring that questions related to the accuracy or integrity of any part of the work are appropriately investigated and resolved.

## Conflict of Interest

The author declares that the research was conducted in the absence of any commercial or financial relationships that could be construed as a potential conflict of interest.
